# A comparative study of the elastic fibre system within the mouse and human cornea

**DOI:** 10.1016/j.exer.2018.07.024

**Published:** 2018-12

**Authors:** Eleanor M. Feneck, Philip N. Lewis, Jim Ralphs, Keith M. Meek

**Affiliations:** a Structural Biophysics Research Group, School of Optometry and Vision Sciences, Cardiff University, Maindy Road, Cardiff, CF24 4HQ, UK; b School of Biosciences, Cardiff University, Cathays Park, Cardiff, CF10 3AX, UK

**Keywords:** Elastic fibres, Elastin, Fibrillin-1, Cornea, Trabecular meshwork, Glaucoma, TGF-β, Transforming growth factor beta, IOP, Intraocular pressure, TPEM, Two-photon excitation microscopy, MAGP, Microfibril-associated glycoproteins, SBF-SEM, Serial block face scanning electron microscopy, TEM, Transmission electron microscopy, PBST, phosphate buffered saline tween-20, LOX, Lysyl oxidase, POAG, Primary open angle glaucoma, MFS, Marfan syndrome

## Abstract

The cornea relies on its organised extracellular matrix for maintaining transparency and biomechanical strength. Studies have identified an elastic fibre system within the human posterior cornea, thought to allow for slight deformations in response to internal pressure fluctuations within the eye. However, the type of elastic fibres that exist within the cornea and their roles remain elusive. The aim of this study was to compare the distribution and organisation of the elastic fibres within the posterior peripheral mouse and human cornea, and elucidate how these fibres integrate with the trabecular meshwork, whilst characterising the distribution of their main likely components (fibrillin-1, elastin and type VI collagen) in different parts of the cornea and adjacent sclera.

We identified key differences in the elastic fibre system between the human and mouse cornea. True elastic fibres (containing elastin) were identified within the human posterior peripheral cornea. Elastic fibres appeared to present as an extensive network throughout the mouse corneal stroma, but as fibrillin-rich microfibril bundles rather than true elastic fibres. However, tropoelastin staining indicated the possibility that true elastic fibres had yet to develop in the young mice studied. Differences were also apparent within the anatomy of the trabecular meshwork. The human trabecular meshwork appeared to insert between the corneal stroma and Descemet's membrane, with elastic fibres continuing into the stroma from the trabecular meshwork anterior to Descemet's membrane. Within the mouse cornea, no clear insertion point of the trabecular meshwork was seen, instead the elastic fibres within the trabecular meshwork continued into Descemet's membrane, with the trabecular meshwork joining posterior to Descemet's membrane.

## Highlights

• True Elastic fibres (containing elastin) occur within the human posterior peripheral cornea.
• A fibrillin-rich microfibril system was found throughout the mouse corneal stroma, with no apparent true elastic fibres.
• Human trabecular meshwork inserts between the posterior corneal stroma and Descemet's membrane.
• A continuity of elastic fibres was identified between cornea and trabecular meshwork in both species.

## 1 Introduction

The cornea enables vision by providing two-thirds of the eye's refractive power (Ganguli et al., 1975). The precise organisation and regulation of extracellular matrix components controls corneal function. The spatial arrangement of the constituent collagen fibrils and the subsequent organisation of the collagen fibrils into lamellae within the stroma provide the properties of transparency and biomechanical strength respectively (Meek and Knupp, 2015). Interfibrillar proteoglycans have also been well documented to maintain corneal physiology by regulating collagen fibril spacing (Rada et al., 1993).

Elastic fibres (which are essentially bundles of fibrillin-rich microfibrils with or without an amorphous central core of elastin) have specific functions in different tissues, but their main role includes providing elastic properties, whilst regulating the bioavailability of transforming growth factor β (TGF-β) (Baldwin et al., 2013). These elastic properties allow tissues to deform and return to their original configuration when subjected to external forces (Kielty et al., 2002). Recently, there has been much controversy within the literature concerning elastic fibres within the cornea, even though their existence was first detected in the mid-19th century (Kolliker, 1860). The presence of elastic fibres was overlooked for many years until the advancement of imaging techniques led to microfibrils being imaged in the cornea throughout different animal species (Alexander and Garner, 1983; Bruns et al., 1987). Even though studies were beginning to image microfibrils within the cornea at a high resolution across different species, their presence within the adult human cornea was disputed. More recent studies have identified an extensive elastic fibre system concentrated within the human posterior peripheral cornea using two-photon excitation fluorescence (TPEM) as well as tannic acid and orcein-based staining methods, both well documented to stain for elastic fibres in electron microscopy (Hanlon et al., 2015; Kamma-Lorger et al., 2010; Lewis et al., 2016). Elastic fibre abnormalities associated with disrupted corneal architecture in Marfan syndrome and keratoconus have further emphasised the important role elastic fibres likely play in maintaining normal corneal function (White et al. 2017a, 2017b). Despite this, the classification of the type of elastic fibre that is present (i.e the presence or absence of elastin within the fibrillin-rich microfibril bundles) and their precise function within the cornea remains elusive.

Elastic fibres differ in their ratio of elastin and microfibril components, providing tissues with different properties. Oxytalan fibres, the most immature elastic fibre are composed of bundles of fibrillin-rich microfibrils (Fullmer and Lillie, 1958; Sawada et al., 2006). Elaunin fibres mature from oxytalan fibres as an intermediate elastic fibre containing small quantities of amorphous elastin (Carrington et al., 1984). True elastic fibres are the most mature fibre type, containing an amorphous elastin core surrounded by microfibril bundles. True elastic fibres develop from oxytalan fibres, proceeding with the deposition of tropoelastin onto the fibrillin-rich microfibril bundles, followed by the cross-linking of tropoelastin into elastin (Baldwin et al., 2013).

The different components within elastic fibres provide tissues with different properties. Fibrillin-rich microfibril bundles form stable structures for maintaining tissue architecture, whilst modulating TGF-β availability (Sengle and Sakai, 2015). Dysfunction of the glycoprotein fibrillin-1 is seen within the autosomal dominant genetic disease, Marfan syndrome. This condition has been seen to result in a thinner and less curved cornea, indicating that functional fibrillin glycoproteins maintain corneal shape (White et al., 2017a). The amorphous elastin component of true elastic fibres permits elasticity, allowing tissues to return to their original shape after deformation (Debelle and Tamburro, 1999; Green et al., 2014). Many components have been shown present within the elastin-microfibril interface or associating with the elastic fibre-cell interface (Kielty et al., 2002). Microfibril-associated glycoproteins (MAGPs) have been identified to contribute to elastic fibre formation, as well as mediating type VI collagen interactions with fibrillin microfibrils, where type VI collagen is thought to anchor true elastic fibres into the extracellular matrix (Gibson et al., 1998). In addition, proteoglycans including decorin and biglycan have been shown to interact with tropoelastin and MAGPs in elastic fibre development (Reinboth et al., 2002). The variety of components found associated with elastic fibres indicates the complexity of the function of the elastic fibre network.

Alexander and Garner were one of the first to identify oxytalan fibres within the cornea using histological staining techniques (Alexander and Garner, 1983). More recent studies have used two-photon fluorescence and electron microscopy to describe the elastic fibre distribution within the cornea. These techniques detect elastin and fibrillin simultaneously, therefore, distinguishing between these proteins to classify the type of elastic fibres that are present has proven a challenge (Kamma-Lorger et al., 2010; Lewis et al., 2016). Identifying the type of elastic fibres that exist within the cornea using antibodies that specifically label the elastic fibre proteins is necessary to elucidate their roles and biomechanical function in the ocular system (Umihira et al., 1994).

To conserve normal vision, aqueous humor flows against resistance through the trabecular meshwork to maintain a physiological intraocular pressure (IOP). A disruption to the outflow of aqueous humor can lead to an increased IOP, a contributing risk factor for glaucoma progression, potentially resulting in blindness (Carreon et al., 2017). The elastic fibre system within the ciliary body tendons of the trabecular meshwork has been shown to merge with the pre-Descemet's layer of the cornea (Dua et al., 2013; Marando et al., 2017; Park et al., 2016). High magnification electron microscopy imaging has also determined that the human trabecular meshwork inserts between Descemet's membrane and the posterior corneal stroma 250 μm from Descemet's membrane termination (Lewis et al., 2016). The interaction of these structures may manipulate aqueous humor outflow to maintain a physiological IOP.

To our knowledge, this study is the first to identify true elastic fibres within the human posterior peripheral cornea using immunofluorescence, specifically labelling elastin and fibrillin-1. In addition, this study has identified key differences within the elastic fibre distribution and trabecular meshwork anatomy between the human and mouse cornea using serial block face-scanning electron microscopy (SBF-SEM), transmission electron microscopy (TEM) and two-photon excitation microscopy (TPEM).

## 2 Methods

### 2.1 Tissue collection

10 mice (Charles Rivers, C57BL/6) were sacrificed following humane schedule one killing methods at 9 weeks old. 12 mouse eyes were fixed in Karnovsky's fixative for 3 h at 4 °C (Graham and Karnovsky, 1966). 8 mouse eyes were frozen on dry ice and cryosectioned transversely at 10 μm thickness using a Leica CM3050 S cryostat, collecting sections on Superfrost Plus Slides (Thermo Scientific, UK).

Eight human corneas containing the scleral ring were obtained from NHS Blood and Transplant (NHSBT). Cornea 1, from a 50 year old male, was dissected into quadrants and immersed in Karnovsky's fixative for 3 h at 4 °C and prepared for electron microscopy as below. Cornea 2 was from a 31-year old male, corneas 3 and 4 were from a 66-year old male, corneas 5 and 6 were from a 69 year old male, cornea 7 was from a 77 year old female and cornea 8 was from a 75 year old male. Cornea 2, 3, 4, 5, 6, 7 and 8 were dissected into quadrants, frozen on dry ice and cryosectioned transversely at 10 μm thickness using a Leica CM3050 S cryostat, collecting sections on Superfrost Plus Slides (Thermo Scientific, UK).

### 2.2 Electron microscopy

Elastic fibres were stained with tannic acid-uranyl acetate (Simmons and Avery, 1980). Karnovsky's fixed quadrants were washed in sodium cacodylate buffer 3 times over 10 min and in distilled water (dH₂0) for 5 min. Samples were post-fixed in 1% osmium tetroxide for 1 h, washed with dH₂0 3 times over 20 min before being transferred to 0.5% filtered tannic acid (TA) in dH₂0 for 2 h. Samples were washed with dH₂0 3 times over 30 min and left overnight in 2% aqueous uranyl acetate (UA). Samples were then dehydrated in a 70–100% ethanol series. Samples were further en bloc stained with 1% UA for 2 h, followed by lead acetate in 1:1 ethanol and acetone for 2 h. The samples were washed with 1:1 ethanol acetone twice over 20 min and then washed 3 times over 20 min with 100% acetone. Samples were infiltrated with 1:1 acetone and araldite resin (araldite monomer CY212 and DDSA hardener) for 1 h. BDMA accelerator was added to the pre-made araldite resin, making continuous resin changes to the samples every 2 h until 6 changes had been made. The samples were embedded and polymerised at 60 °C for 48 h.

#### 2.2.1 Serial-block face scanning electron microscopy

Samples were mounted onto a Gatan specimen pin and coated with silver conductive epoxy adhesive (TAAB laboratories). The pin was sputtered with gold and placed inside the Zeiss Sigma VP FEG SEM equipped with a Gatan 3View system. Automated serial sectioning was undertaken of the block face surface every 50 nm. A dataset of 1000 images was acquired. 3-D reconstructions of the datasets were produced using either the manual segmentation function (for larger cell and membrane structures) or the automatic isosurface function (for the finer elastic fibre network where manual segmentation was not practical) using Amira 6.4 software (FEI, Mérignac, France).

#### 2.2.2 Transmission electron microscopy

Ultrathin sections were cut (90 nm) of the same blocks at the end of the serial sectioning using the Leica UC6 ultra-microtome, collected on 300 hexagonal copper grids and analysed using the JEOL 1010 transmission electron microscope (TEM).

### 2.3 Two-photon excitation microscopy

Cover slips were added to cryosections and they were imaged using the LSM 510 META NLO upright multi-photon laser scanning microscope (Carl Zeiss), equipped with Zen Software. The laser was excited at 800 nm with a main beam splitter at 690 to reject the light longer than the specified wavelength. A bandpass filter was used at 505/119 bandwidth to collect the elastic fibre auto-fluorescent signal. Images were recorded at ×20 or ×40 magnifications.

### 2.4 Immunofluorescence

The cryosections were circumscribed with a water repellent delimiting pen (ImmEdge Hydrophobic Barrier PAP pen, Vector labs) before being rehydrated with phosphate buffered saline solution (PBST [Tween-20, 0.1% Tween-20, 0.05 M, pH 7.3]). Cryosections were blocked with 5% horse serum in PBST for 20 min (Levy, 1980). Primary antibodies were added to the cryosections and incubated for 24 h at 4 °C, washed in PBST (3 changes over 10 min) before adding secondary antibodies. Cryosections were incubated for 5 h at room temperature before secondary antibodies were washed off with PBST. Cover slips (VWR International) were added to the cryosections using VECTASHIELD HardSet Antifade Mounting Medium, containing DAPI to label nuclei blue. Cryosections were imaged using the Olympus BX61 epifluorescence microscope, equipped with an F-view Digital camera using ×10,×20 and ×40 objectives.

#### 2.4.1 Antibodies

Rabbit polyclonal (Elastin, Tropoelastin, Type VI collagen) and mouse monoclonal (Fibrillin-1) primary antibodies were used (Abcam) (Guillen-Ahlers et al., 2008; Mondrinos et al., 2007; Pan et al., 2015; Raghavan et al., 2009). Dylight 594 Horse Anti-Mouse IgG, Dylight 488 Horse Anti-Rabbit IgG and Dylight 594 Horse Anti-Rabbit were applied as secondary antibodies to the mouse monoclonal and rabbit polyclonal primary antibody treated sections respectively (Vector labs).

## 3 Results

### 3.1 Electron microscopy

#### 3.1.1 Mouse

**Fig. 1:**
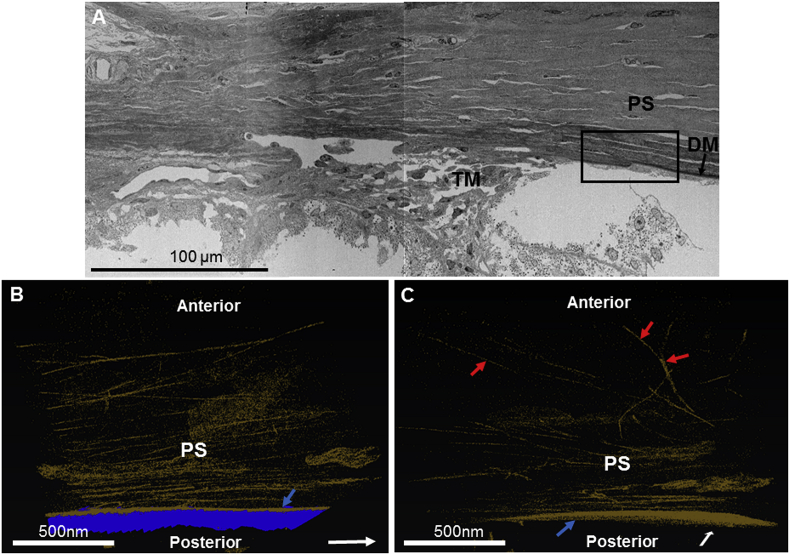
Elastic fibre reconstructions within the mouse peripheral cornea. Fig. 1A: SBF-SEM stitched image of the mouse trabecular meshwork (TM) and posterior peripheral cornea stroma (PS). Higher magnification SBF-SEM imaging within the area of Descemet's membrane (DM) termination (black square) used for reconstructions in Fig. 1B and C. Fig. 1B: Reveals the presence of an elastic fibre system (gold) throughout the posterior peripheral corneal stroma, with a highly stained sheet (blue arrow) of elastic fibres appearing anterior to Descemet's membrane (blue). The white arrow identifies the direction towards the central cornea. Fig. 1C: The 3D dataset rotated 90° to Fig. 1B, shows elastic fibres (gold) occasionally bifurcating and continuing within the same plane (red arrows). The white arrow indicates the direction towards the central cornea. (The three-dimensional human reconstruction can be seen in Supplementary Video 1). (For interpretation of the references to colour in this figure legend, the reader is referred to the Web version of this article.)

**Fig. 2:**
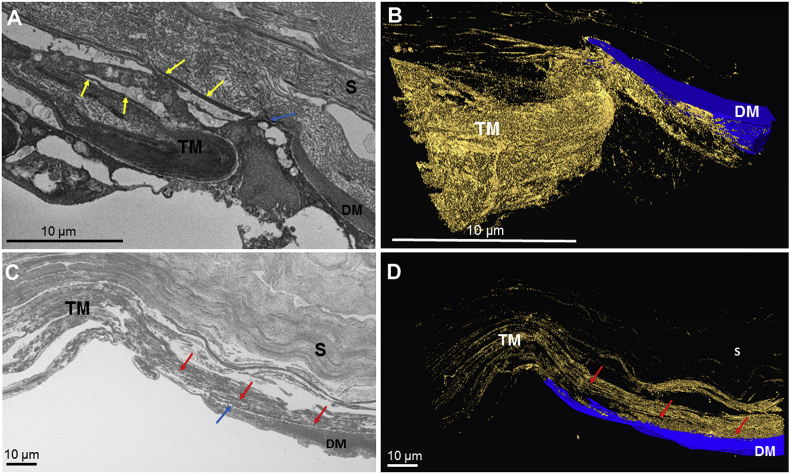
A comparison of the termination of Descemet's membrane within the mouse (Fig. 2A, B) and human cornea (Fig. 2C, D). Fig. 2A: The termination of Descemet's membrane within the mouse cornea appears to run anterior to the trabecular meshwork (blue arrow). Elastic fibres are present within the trabecular meshwork and within the stroma (yellow arrows). Fig. 2B: SBF-SEM 3-D reconstruction of Fig. 2A shows the elastic fibre system (gold) anterior to the trabecular meshwork and Descemet's membrane (blue), with no clear insertion point of the trabecular meshwork into the posterior peripheral cornea. (The three-dimensional video of the mouse reconstruction can be seen in Supplementary Video 2). Fig. 2C: The termination of Descemet's membrane (blue arrow) within the human cornea runs posterior to the trabecular meshwork (TM), with the trabecular meshwork appearing to insert within the posterior peripheral corneal stroma (red arrows). Fig. 2D: SBF-SEM 3D reconstruction of Fig 2C reveals the elastic fibre system (gold) in the trabecular meshwork (TM) continuing anterior to Descemet's membrane (blue), within the corneal stroma (S). These fibres insert between the posterior peripheral cornea stroma (S) and Descemet's membrane (DM). (The three-dimensional human reconstruction can be seen in Supplementary Video 3). (For interpretation of the references to colour in this figure legend, the reader is referred to the Web version of this article.)

**Fig. 3:**
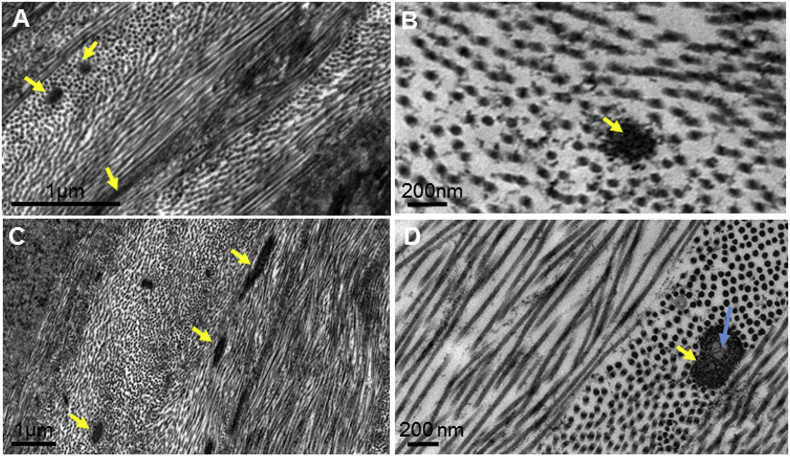
Transmission electron microscope images of tannic acid stained elastic fibres (yellow arrow) within the mouse cornea (Fig. 3A, 3B) and human cornea (Fig. 3C, 3D). Fig. 3A: Fibres within the posterior peripheral cornea in oblique or longitudinal section, indicating their different directions within the plane of the cornea. Fig. 3B: High magnification cross-section of a mouse elastic fibre showing no clear amorphous core, indicating that they are bundles of fibrillin-rich microfibrils, rather than elastin-containing elastic fibres. Fig. 3C: Fibres within the human posterior peripheral cornea in oblique or longitudinal section. Fig. 3D: High magnification image of a human elastic fibre cross-section within the peripheral cornea showing a clear amorphous core surrounded by microfibrils. (For interpretation of the references to colour in this figure legend, the reader is referred to the Web version of this article.)

The tannic acid staining method revealed an extensive elastic fibre system within the mouse peripheral corneal stroma and trabecular meshwork (Fig. 1). Reconstructions indicated a concentrated elastic fibre sheet anterior to Descemet's membrane, with individual elastic fibres presenting throughout the corneal stroma, running longitudinally and transversely between collagen lamellae (Fig. 1B–C). The elastic fibres associated with Descemet's membrane integrated with those within the trabecular meshwork, representing a continuous fibre system (Fig. 2 A–B). Descemet's membrane terminated anterior to the trabecular meshwork, with the trabecular meshwork elastic fibre system appearing to merge into Descemet's membrane (Fig. 2 A–B). TEM images revealed elastic fibres with no apparent central amorphous components, suggesting they are microfibril bundles (Fig. 3A–C).

#### 3.1.2 Human

The tannic acid staining method identified elastic fibres concentrated anterior to Descemet's membrane, appearing to continue from the trabecular meshwork (Fig. 2). The elastic fibres within the trabecular meshwork appeared to insert between the posterior corneal stroma and Descemet's membrane (Fig. 2C-D). High magnification TEM analysis revealed the elastic fibres to contain an amorphous core surrounded by microfibrils, representing true elastic fibres (Fig. 3C–D).

### 3.2 Two-photon excitation microscopy

#### 3.2.1 Mouse

**Fig. 4:**
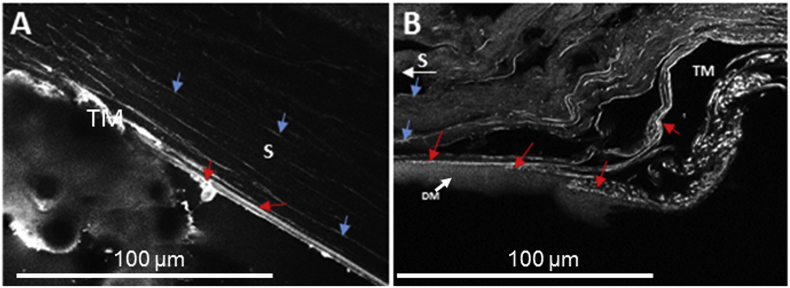
Two-photon-excitation microscopy images with the microscope tuned to fluoresce elastic fibres within the mouse (Fig. A) and human (cornea 2) peripheral cornea (Fig. B) and Descemet's membrane termination area. Fig. 4A: Auto-fluorescence appeared throughout the mouse corneal stroma as longitudinal fibres running parallel to the corneal surface (blue arrows). A decreased signal was seen within the central cornea. The signal was strongest within the trabecular meshwork, appearing to continue with Descemet's membrane (red arrows). Fig. 4B: Auto-fluorescence expressed strongest within the human trabecular meshwork (TM), with a continuation of expression running anterior to Descemet's membrane (DM) (red arrows) and further continued into the posterior peripheral corneal stroma (S). White arrow indicates direction towards the central cornea. (For interpretation of the references to colour in this figure legend, the reader is referred to the Web version of this article.)

TPEM identified the auto-fluorescent signal from elastic fibres running within the plane of the corneal stroma. An increased expression appeared within the trabecular meshwork, continuing to run anterior to Descemet's membrane within the peripheral cornea. The expression appeared to decrease within the central cornea, however, fibres still appeared to be auto-fluorescing throughout the corneal stroma (Fig. 4A).

#### 3.2.2 Human

TPEM identified elastic fibres within the trabecular meshwork and posterior peripheral cornea, with decreased signal within the central cornea (Fig. 4B). A continuation of auto-fluorescence was detected from the trabecular meshwork into the peripheral corneal stroma and anterior to Descemet's membrane, with the trabecular meshwork fibres appearing to insert between Descemet's membrane and the corneal stroma (Fig. 4B).

### 3.3 Immunofluorescence

Antibodies to type VI collagen, elastin, tropoelastin and fibrillin-1 were used to label elastic fibre-associated proteins. All controls for the immunofluorescent results showed no background staining, indicating that all positive staining imaged is associated with the primary antibody applied (Fig. 8. Appendix).

#### 3.3.1 Mouse

**Fig. 5:**
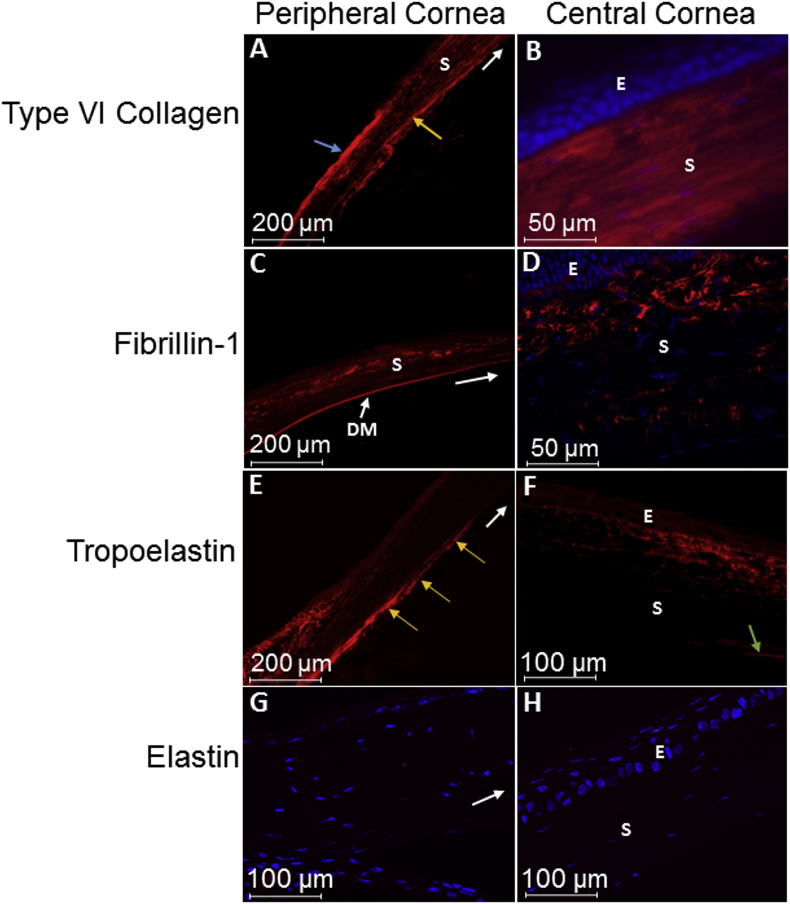
Mouse cornea immunofluorescence. DAPI staining in blue represents cell nuclei; E: epithelium; s: stroma; DM: Descemet's membrane. Fig 5A and 5B: Type VI collagen appeared to express throughout the corneal stroma, with enhanced expression within the anterior sclera (blue arrow) and posterior peripheral cornea (yellow arrow). Fig. 5C and 5D: Fibrillin-1 was expressed throughout the corneal stroma, appearing enhanced within the anterior stroma and Descemet's membrane. Fig. 5E: Tropoelastin was strongly expressed within the posterior peripheral cornea (yellow arrows). Fig. 5F: Tropoelastin also showed expression within the anterior central corneal stroma, however, staining in both the posterior cornea terminated before reaching the central cornea (c) (green arrow). Fig. 5G and 5H: Elastin failed to express throughout the mouse cornea. White arrows indicate the direction towards the central cornea. (For interpretation of the references to colour in this figure legend, the reader is referred to the Web version of this article.)

Type VI collagen was present throughout the corneal stroma, appearing enhanced within the posterior peripheral corneal stroma (Fig. 5A and B). Fibrillin-1 occurred throughout the corneal stroma of the mouse model as fibres, appearing enhanced within the anterior stroma (Fig. 5C and D). Elastin expression was negative within the mouse cornea, however, tropoelastin appeared highly expressed within the posterior peripheral cornea and within the anterior central corneal stroma (Fig. 5E–H).

#### 3.3.2 Human

**Fig. 6:**
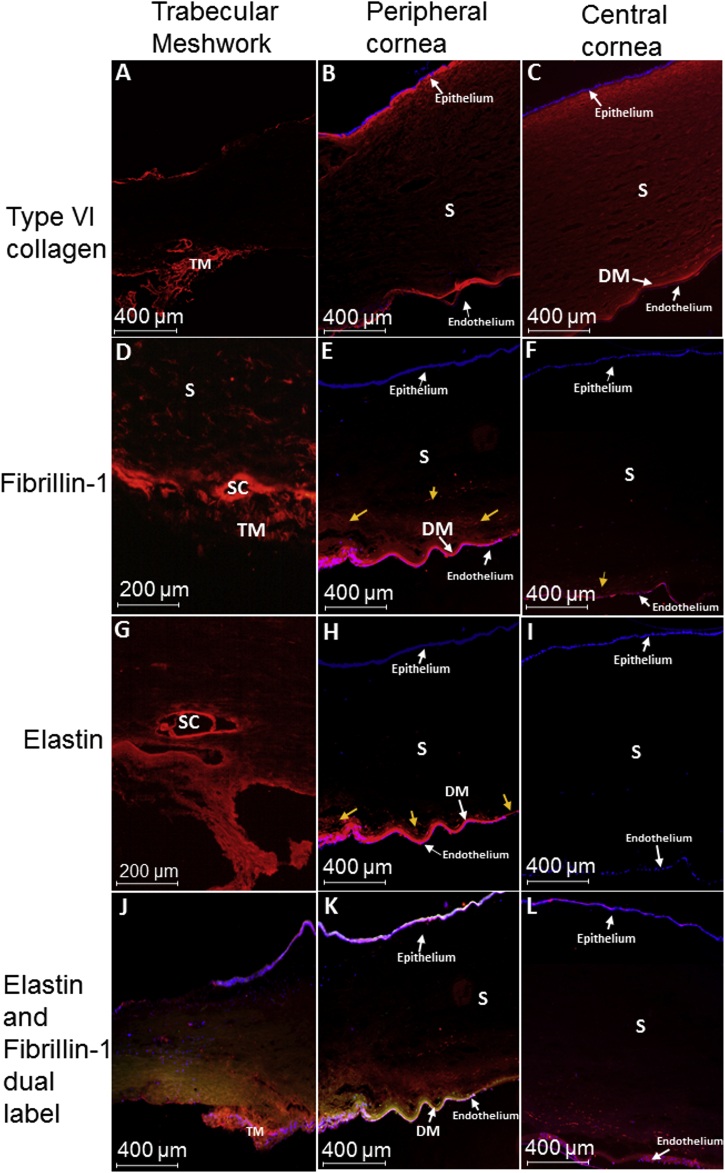
Human cornea immunofluorescence, results shown from cornea 2 (A & D), cornea 4 (G), cornea 6 (B & C), cornea 7 (E, F, H–L). All corneal tissues showed the same results, except for a small expression of fibrillin-1 directly under Bowman's membrane within the anterior cornea of corneas 3 and 4 (results not shown). Fig. 6 A–C: Type VI collagen expressed within the trabecular meshwork and corneal stroma of all corneal tissue analysed. Fig. 6 D–F: Fibrillin-1 expression appeared to be increased within the trabecular meshwork and posterior peripheral cornea. Expression appeared to decrease travelling towards the central cornea. Fibrillin-1 expression showed similar results across all corneal tissue, however, in addition, expression presented in the anterior corneal stroma directly inferior to Bowman's membrane in 2 corneas taken from the same donor (corneas 3 and 4), this was not seen in any other corneal tissue analysed. Fig. 6 G–I: Elastin expression within all three corneal tissues presented within the trabecular meshwork and posterior peripheral cornea, appearing enhanced anterior to Descemet's membrane (DM). No elastin expression presented within the central or anterior corneal stroma. Fig 6 J–L: Dual labelling shows elastin (green) and fibrillin-1 (red) co-localising (orange) within the TM and peripheral cornea. Fibrillin-1 is found present without elastin within the central cornea. S: stroma; TM: trabecular meshwork; SC: Schlemm's canal; DM: Descemet's membrane. (For interpretation of the references to colour in this figure legend, the reader is referred to the Web version of this article.)

Type VI collagen was expressed throughout the corneal stroma, appearing enhanced within the trabecular meshwork and posterior peripheral cornea (Fig. 6A–C). Fibrillin-1 was expressed within the trabecular meshwork and peripheral cornea (Fig. 6D–F). Fibrillin-1 staining continued anterior to Descemet's membrane, appearing to decrease towards the central cornea, with little expression within the posterior stroma of the central cornea (Fig. 6F). Elastin staining was increased within the trabecular meshwork and peripheral posterior cornea of all corneas analysed (Fig. 6G–H). Elastin expression continued from the trabecular meshwork, anterior to Descemet's membrane, with no expression within the central cornea (Fig. 6G–I). Tropoelastin expression was negative within the human corneal tissue analysed, indicating that all tropoelastin has been cross-linked to become elastin (results not shown). Dual labelling of fibrillin-1 and elastin showed co-localisation of the proteins in the trabecular meshwork and peripheral posterior cornea (Fig. 6J–L).

## 4 Discussion

This study compared the elastic fibre system as well as the trabecular meshwork insertion points between the mouse and human cornea, classifying the type of elastic fibres present. Tannic acid has previously been identified to stain components of true elastic fibres and microfibrils, including both the amorphous elastin core and the surrounding fibrillin-rich microfibrils (Lewis et al., 2016; Simmons and Avery, 1980). Mouse cornea SBF-SEM reconstructions of the elastic fibre system revealed a concentrated network of elastic tissue directly anterior to Descemet's membrane, with individual elastic fibres presenting throughout the corneal stroma. The elastic fibre system was more extensive in the mouse cornea compared to previous reconstructions of the human elastic fibre system. Elastic fibres within the mouse cornea were shown throughout the corneal stroma, whereas within the human cornea elastic fibres are abundant in the posterior 200 μm stroma (Lewis et al., 2016). Transmission electron microscopy indicated that the mouse elastic fibre system is mainly composed of fibrillin-rich microfibril bundles, with no apparent elastin amorphous core. Within the human posterior peripheral cornea, elastic fibres showed an amorphous elastic core surrounded by microfibrils, indicating the presence of true elastic fibres. An amorphous core was also seen in previous studies analysing the posterior peripheral human elastic fibres (Lewis et al., 2016).

**Fig. 7:**
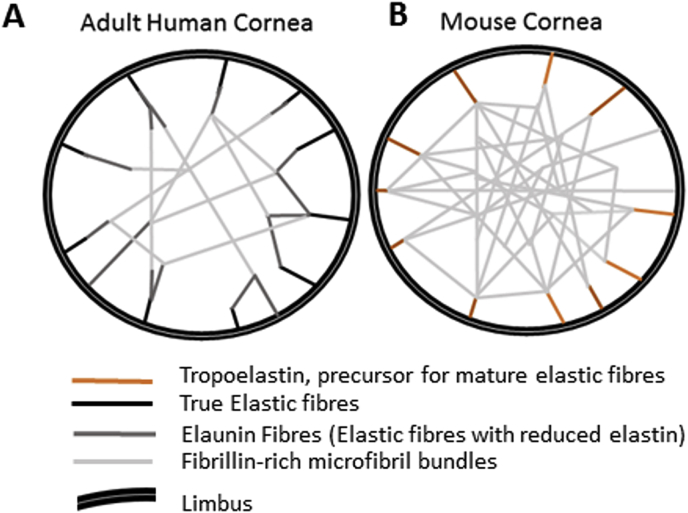
Proposed elastic fibre system within the human cornea (**A**) and mouse cornea (**B**). The results from this study appear to confirm true elastic fibres within the human posterior peripheral cornea, potentially providing elastic properties, allowing slight deformations within the peripheral cornea. A decreased presence of elastin with fibrillin-1 expression indicated a gradual transition from true elastic fibres via elaunin to fibrillin-rich microfibril bundles) towards the central cornea. Fibrillin-rich microfibrils provide support to the central cornea, whilst elastin is not required as deformations to the central cornea would disrupt vision. Within the mouse cornea no elastin was detected, indicating no true elastic fibres. However, tropoelastin expression indicates a possibility of elastin formation when required, most probably during wound healing. The fibrillin-1 expression was enhanced throughout the mouse cornea.

**Fig. 8:**
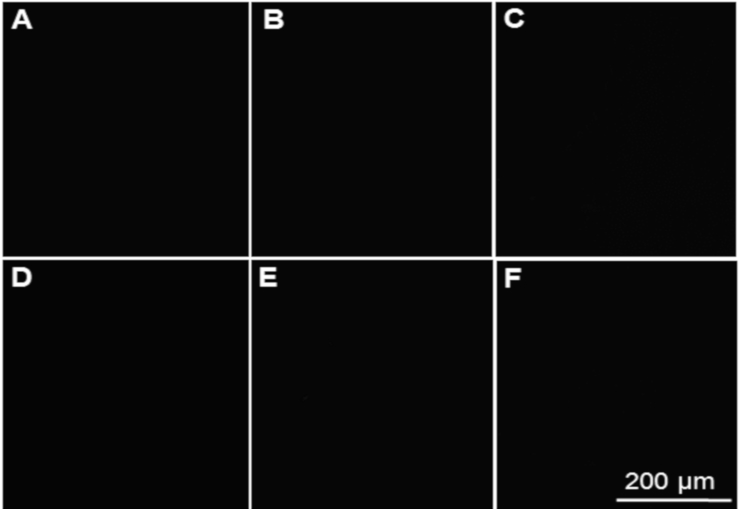
Immunofluorescence controls all appeared to show no apparent staining. All controls taken with a 500 ms exposure time, the same exposure time given to all of the stained slides. All controls were also captured at a ×10 magnification Fig. 8A: Mouse control containing no primary antibody and an anti-mouse secondary antibody Fig. 8B: Mouse control containing mouse immunoglobulin and subsequent anti-mouse secondary antibody. Fig. 8C. Mouse control containing rabbit immunoglobulin and anti-rabbit secondary antibody. Fig. 8D. Human control containing no primary antibody with anti-rabbit secondary antibody. Fig. 8E. Human control containing rabbit immunoglobulin and anti-rabbit secondary antibody. Fig. 8F. Human control containing mouse immunoglobulin with anti-mouse secondary antibody.

TPEM detected elastic fibres throughout the mouse corneal stroma and within the trabecular meshwork and posterior peripheral cornea of the human cornea. Even though TPEM is a documented technique for elastic fibre verification, it cannot distinguish between elastin and fibrillin components (Mansfield et al., 2009). To further classify the type of elastic fibre, localised immunofluorescence was used and true elastic fibres were identified within the posterior peripheral human cornea. Elastin and fibrillin-1 stained within the posterior peripheral corneal stroma and directly anterior to Descemet's membrane, extending from the sclera and trabecular meshwork. Elastin was not expressed within the central cornea, whilst fibrillin-1 was, indicating the presence of elastin-free microfibril bundles in the central cornea. The results generated from this study allow us to suggest models for the elastic fibre composition across the human and mouse corneas (Fig. 7). Differences were identified within the mouse cornea, with an enhanced fibrillin-1 staining profile throughout the corneal stroma. Elastin failed to positively express within the mouse tissue, despite this, tropoelastin expression indicates the possibility of elastic fibre assembly. Older mouse tissue should be analysed to determine if the tropoelastin eventually becomes cross-linked to form true elastic fibres. However, mice may not live long enough for true elastic fibres to be necessary within the cornea. The negative staining for tropoelastin within the human cornea illustrates that all of the tropoelastin has been cross-linked into true elastic fibres by the age of 31 years within the human cornea; to determine when maturation of tropoelastin is complete, analysis of younger corneal tissue would be required.

Deformations have been described within the peripheral cornea in response to the intraocular pulse, allowing the cornea to move forward, whilst the central cornea maintains its shape (Boyce et al., 2008; White et al., 2017b). The elastin component of true elastic fibres would permit slight deformations within the peripheral cornea. Their presence within the peripheral cornea may also oppose IOP forces to prevent the cornea from bulging outwards. Due to the structural support fibrillin molecules provide throughout biological systems, their presence is hypothesised to add reinforcement in compliant tissues (Sherratt et al., 2003). In addition, fibrillin microfibrils may hold the ability to extend more than collagen fibrils, providing a secondary line of defence should the collagen start to rupture.

In keratoconus, additional microfibrils are laid down within the anterior human corneal stroma, and are thought to provide strength to prevent corneal rupturing (White et al., 2017b). Lysyl oxidase (LOX) is significantly decreased within keratoconus as well as in conditions including Down's syndrome, where the prevalence of keratoconus is enhanced (Dudakova and Jirsova, 2013). The elastic fibre system within the central cornea in keratoconus has recently been shown to be disrupted, with microfibrils presenting within the anterior stroma (White et al., 2017b). The reduced LOX availability would reduce the tropoelastin cross-linking that is required for true elastic fibre assembly, so it is likely that this would affect the presence of true elastic fibres in the peripheral keratoconus cornea also. Without true elastic fibres, elastic deformation would not be confined to the peripheral cornea, corneal shape would be disrupted and this could result in corneal ectasia. The increased microfibril deposition within the anterior stroma in keratoconus may thus compensate for the loss of true elastic fibres within the periphery, providing additional support for the central cornea. Unfortunately, this hypothesis is difficult to test as peripheral corneal tissue from keratoconus patients is rarely available for study.

Fibrillin-rich microfibrils were expressed throughout the corneal stroma of the mouse cornea, with enhanced expression within the anterior corneal stroma. Due to the lack of elastin within the mouse cornea, this microfibril system could provide structural support to the cornea to help maintain corneal structural integrity.

Corneal structure and function is significantly disrupted in Marfan syndrome (MFS). This condition results from mutations in the FBN1 gene encoding fibrillin-1, disrupting true elastic fibre assembly and the normal biomechanical functioning of tissues (Hollister et al., 1990). The cornea is significantly thinner and less curved in MFS, indicating that functional microfibrils are required for maintaining corneal structure (Gehle et al., 2017; White et al., 2017a). Their enhancement where the cornea is vulnerable confirms their importance in providing structural support. Primary open angle glaucoma (POAG) prevalence is significantly increased within Marfan syndrome, indicating that disruptions to the elastic fibre system lead to enhanced IOP and aqueous humor outflow resistance (Izquierdo et al., 1992). Elastin synthesis and fibrillin regulated TGF-β signalling is also increased in patients with POAG, directly impacting the trabecular meshwork, indicating that the elastic fibre system maintains a physiological cornea and aqueous humor outflow (Han et al., 2011; Umihira et al., 1994). Three-dimensional SBF-SEM reconstructions within the mouse cornea indicated a continuation of the elastic fibre system between the trabecular meshwork and peripheral cornea, with no clear insertion point into the corneal stroma. This contrasts with reconstructions from the same region in the human cornea, where the trabecular meshwork inserts between the posterior peripheral corneal stroma and Descemet's membrane 250 μm after Descemet's membrane termination (Lewis et al., 2016). This anatomical difference between the mouse and human could indicate an evolutionary advance of the human cornea in regulating IOP outflow and corneal physiology. However, the continuation of the elastic fibre system occurs in both models, indicating that the elastic fibre system possesses an important function between the cornea and the trabecular meshwork. The elastic fibres may anchor the trabecular meshwork into the cornea, holding it taut. If so, this system may be necessary to maintain normal IOP and corneal structure. The elastic fibre system could provide a potential target for treatment strategies for glaucoma, but more research is needed to determine the role of elastic fibres in the trabecular meshwork and peripheral cornea.

In conclusion, this study has identified true elastic fibres within the human posterior peripheral cornea. True elastic fibres were not seen within the central cornea, indicating that their importance only lies within the periphery. A system of fibrillin-rich microfibril bundles was identified within the central cornea, which is thought to support the collagen in the maintenance of corneal shape and biomechanical strength. The fibrillin-rich microfibril bundles appeared more extensive within the mouse model, this enhancement may provide additional support to prevent the cornea bulging outwards. The mouse also displayed anatomical differences, showing no clear insertion point of the trabecular meshwork between the corneal stroma and Descemet's membrane. The differences identified within the elastic fibre system and trabecular meshwork should be considered when using the mouse model within trabecular meshwork outflow, glaucoma and elastic fibre studies.

## Financial disclosure

No Author has a financial or proprietary interest in any material or method mentioned.

## Conflicts of interest

The Authors have no conflicts of interest to disclose.

## Appendix A Supplementary data

Video Clip S1 Related to Fig. 1. Rendered three dimensional video of the elastic fibre system within the mouse posterior peripheral cornea stroma. Video Clip S1

Video Clip S2 Related to Fig. 2. Rendered three dimensional video of the termination of Descemet's membrane within the mouse cornea. Video Clip S2

Video Clip S3 Related to Fig. 2. Rendered three dimensional video of the termination of Descemet's membrane within the human cornea. Video Clip S3
